# Establishing correlations between normal pancreatic and submandibular gland ducts

**DOI:** 10.1186/s12876-022-02443-2

**Published:** 2022-07-29

**Authors:** Bojan V. Stimec, Dejan Ignjatovic, Johannes A. Lobrinus

**Affiliations:** 1 Anatomy Sector, Teaching Unit, Faculty of Medicine, University of Geneva, Rue Michel-Servet 1, 1211 Geneva, Switzerland; 2 Department of Digestive Surgery, Akershus University Hospital, University of Oslo, 1478 Lorenskog, Norway; 3 Institute of Clinical Medicine, University of Oslo, Blindern, P.O. Box 1171, 0318 Oslo, Norway; 4 Department of Clinical Pathology, Geneva University Hospitals, C.M.U., Rue Michel-Servet 1, 1206 Geneva, Switzerland

**Keywords:** Pancreas, Submandibular gland, Duct, Morphometry, Side branch

## Abstract

**Background:**

The objectives of this study were to evaluate the relationship between ductal morphometry and ramification patterns in the submandibular gland and pancreas in order to validate their common fractal dimension.

**Methods:**

X-ray ductography with software-aided morphometry were obtained by injecting barium sulphate in the ducts of post-mortem submandibular gland and pancreas specimens harvested from 42 adult individuals.

**Results:**

Three cases were excluded from the study because of underlying pathology. There was a significant correlation between the length of the main pancreatic duct (MPD) and the intraglandular portion of the right submandibular duct (SMD) (r = 0.3616; *p* = 0.028), and left SMD (r = 0.595; *p* < 0.01), respectively, but their maximal diameters did not correlate (r = 0.139—0.311; *p* > 0.05). Both dimensions of the SMD showed a significant right-left correlation (*p* < 0.05). The number of MPD side branches (mean = 37) correlated with the number of side branches of left SMD, but not with the right one (mean = 9). Tortuosity was observed in 54% of the MPD, 32% of the right SMD, and 24% of the left SMD, with mutual association only between the two salivary glands.

**Conclusions:**

Although the length of intraglandular SMD and MPD correlate, other morphometric ductal features do not, thus suggesting a more complex relationship between the two digestive glands.

## Background

The two digestive glands, submandibular and pancreas, are unalike at first sight. The submandibular gland is a tubuloacinar seromucous gland, responsible for 2/3 of total saliva output in quiescent state. Its planimetric size is approximately 850 mm^2^ [1], composed of a larger superficial and a smaller deep portion. Its excretory duct runs from the superficial through the deep portion, then takes an extraglandular course in the floor of the mouth. On the other hand, the pancreas, with a length of around 15 cm, belongs to the largest digestive glands in the organism. This is a compound lobulated exocrine and endocrine gland, composed arbitrarily of the tail, body, neck, head and the uncinate process. Its main duct runs along the midline of the gland, receiving the lobular affluents in form of a herringbone, and terminates by a short common channel with the common bile duct [2].

On a second glance, the similarities between the submandibular gland and pancreas exist. Their common embryonic origin is the foregut, and the developmental mechanisms involved are budding, i.e. proliferation and evagination of epithelium, followed by organization of lobes within the mesenchyme, and finally formation of the canalicular system through appearance of lumina in buds [3]. These events are closely synchronous for both glands, beginning at Carnegie stage 13 and 14. It has been observed that the stem cell populations deriving from both glandular tissues express analogous phenotypes and properties [4]. The two glands share the same antigens—the family of water channel protein aquaporins [5, 6].

Further, both the pancreatic and the salivary gland tissue present the fractal dimension, i.e. non-integer geometric property expressed through self-semblance in such a manner that a certain pattern repeats itself cyclically at different size levels [7, 8]. From the clinical point of view, one would like to find out whether there is a similar ramification pattern of both glands at a macroscopic scale, in terms of ductal morphology correlation. This is particularly interesting from the aspect of joint involvement of glands in pathology or as an indirect clinical follow-up of pancreas via salivary glands [1, 9, 10]. It has been shown that ductal pathology of salivary glands compatible with chronic sialoadenitis is common in the course of chronic pancreatitis. Identification of such forms can facilitate effective therapeutic intervention, and sialography may be valuable in identifying primary chronic pancreatitis. Therefore, we have carried out a comparative post-mortem ductographic study on the pancreas and the submandibular glands in order to evaluate the macro-morphological and morphometric correlation between the ductal systems of the two glands.

## Material and methods

The investigation was conducted in accordance with Ethical Principles for Medical Research Involving Human Subjects (World Medical Association, Declaration of Helsinki: Ethical Principles for Medical Research) [WMO 2008]. The post-mortem material for the study was sampled at the Institute for Anatomy, Faculty of Medicine, University of Belgrade, under the legislative points of the Law on Health Protection, Chapter on Establishing time and cause of death and autopsy of deceased persons, and Chapter on Procurement of bodies of deceased persons for the purpose of practical medical education [11, 12]. The local health legislature allowed research on donated bodies. The remaining portion of the study (image analysis, morphometry, statistical analysis, etc.) was conducted at the Faculty of Medicine, University of Geneva. As the data did not contain personal identifiers (anonymous biological material), this research did not require an IRB review under federal law (Human Research Act 810.30, HRA).

### Subjects

Fresh specimens *en bloc* were excised during autopsy of 42 adult persons (35 males, 7 females; median age 54 years, mean 53.1 ± 12.1 years), all of local ethnicity. The height and weight of all the subjects was not available, as they do not pertain to the regular body donation protocol. The harvested organs and their surrounding were carefully inspected and palpated. Only cases without macroscopic pathological findings or injuries of the head-neck transition and of the duodeno-pancreas were included. The first excisate included the tongue, base of the mouth with submandibular and sublingual glands, the hyoid bone, pharynx, larynx, thyroid gland and the upper half of the trachea. The submandibular gland was preferred to the parotid because of an easier approach. The second excisate, from the same individual, consisted of the pancreas, duodenum and roots of the mesentery and transverse mesocolon. Therefore, both original excisates included the surrounding structures, whose rigidity prevented artificial deformation of the ductal system. The sublingual caruncle and the ampulla of Vater were cannulated via a thin Venflon catheter. The water-soluble iodine contrast has the disadvantage that it tends to pass quickly into the parenchyma in the postmortem specimens, consequently blurring the image. We have therefore opted for an 80% barium-sulphate suspension which was diluted in formol (in order to be less viscous) and slowly injected under controlled pressure. The injection was halted on first sign of resistance, or when there was acinar opacification of contrast on the surface of the glands. After image acquisition, the excisates were cleared of adjacent tissue and their weight was measured.

### Image acquisition

All the specimens were placed flat on their larger surface (coronal plane). The position of the ductal system was verified by fluoroscopy, assuring that the ductal system is parallel to the image detector, i.e. film. A metal platelet as a parameter of known width was placed in proximity for later calibration on image analysis. Afterwards, they underwent X-ray ductography using the Seldix 550, EI Nis, with the following properties: voltage 42 kV, exposure 2.5–3.2 mAs.

### Image interpretation

**Fig. 1:**
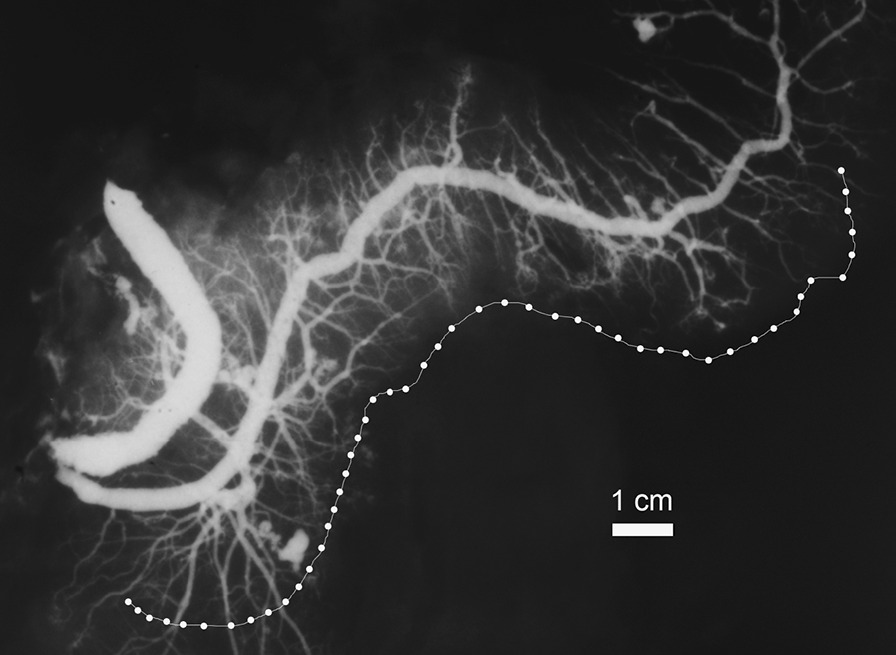
A post-mortem pancreatogram with Freehand contour outline of the MPD below. Note: The Freehand line was drawn following the course of the main duct. For clarity reasons of this illustration, it was afterwards wholly just transposed to the side of the gland, in order not to overlap with the main duct or its side branches

**Fig. 2:**
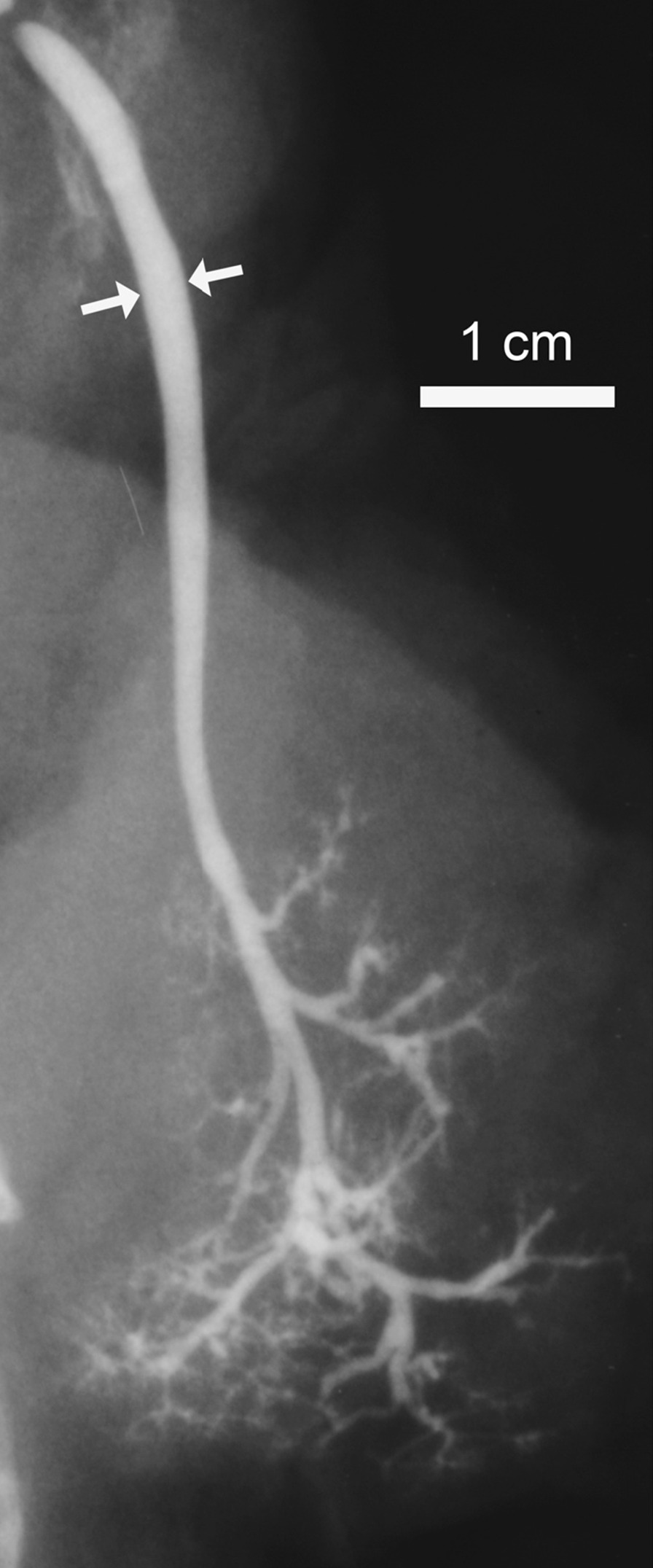
A post-mortem SM sialogram with maximal caliber (arrows)

Once the ductograms were obtained, they underwent detailed morphometric analyses, using the ImageJ, public domain Java image processing software (ImageJ 1.52v, Research Services Branch, National Institute of Mental Health). First, the scale was set according to the known width of the metal platelet. The tools used were Straight line and Freehand line for calibers and lengths, respectively (Figs. 1, 2). The morphometric analyses were carried out by an experienced anatomist and an experienced pathologist. Both authors implied (BVS and JAL) were blinded to the personal data (age, sex) during image interpretation.

The following parameters were analyzed: maximal caliber and total length of the main pancreatic duct (MPD; Wirsung’s duct) and the submandibular duct (SMD, Wharton’s duct), length of the intraglandular SMD, and total number of MPD and SMD side branches. The intraglandular SMD was defined as the largest channel in continuity with the extraglandular SMD, running to the distal pole of the gland. Both the MPD and SMD were analyzed for tortuosities, and the two glands were observed for the existence of accessory lobes or ducts.

### Excluded cases

**Fig. 3:**
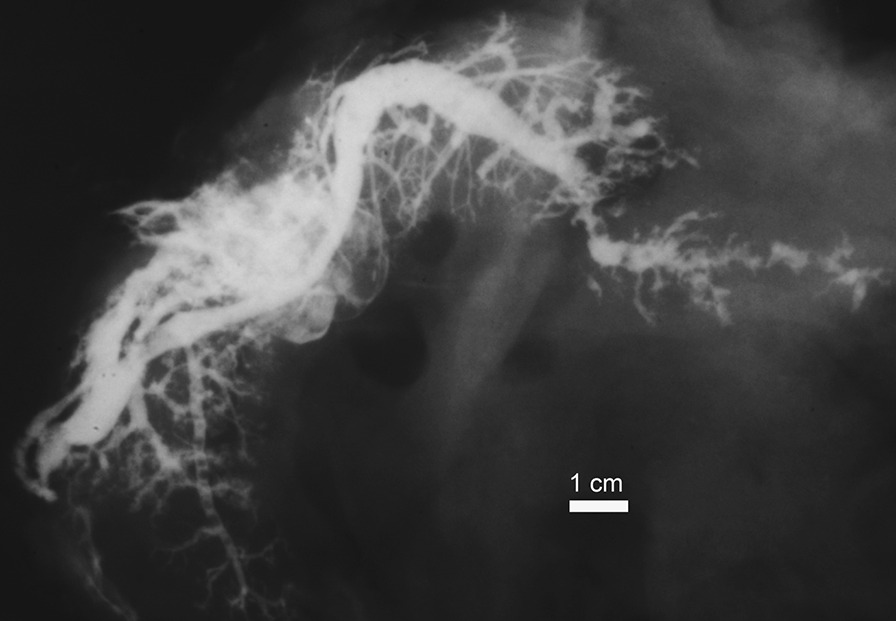
A post-mortem pancreatogram with pathological findings: dilatations and strictures of the MPD, mostly in the body and tail of pancreas; side branches dilatated or obstructed

**Fig. 4:**
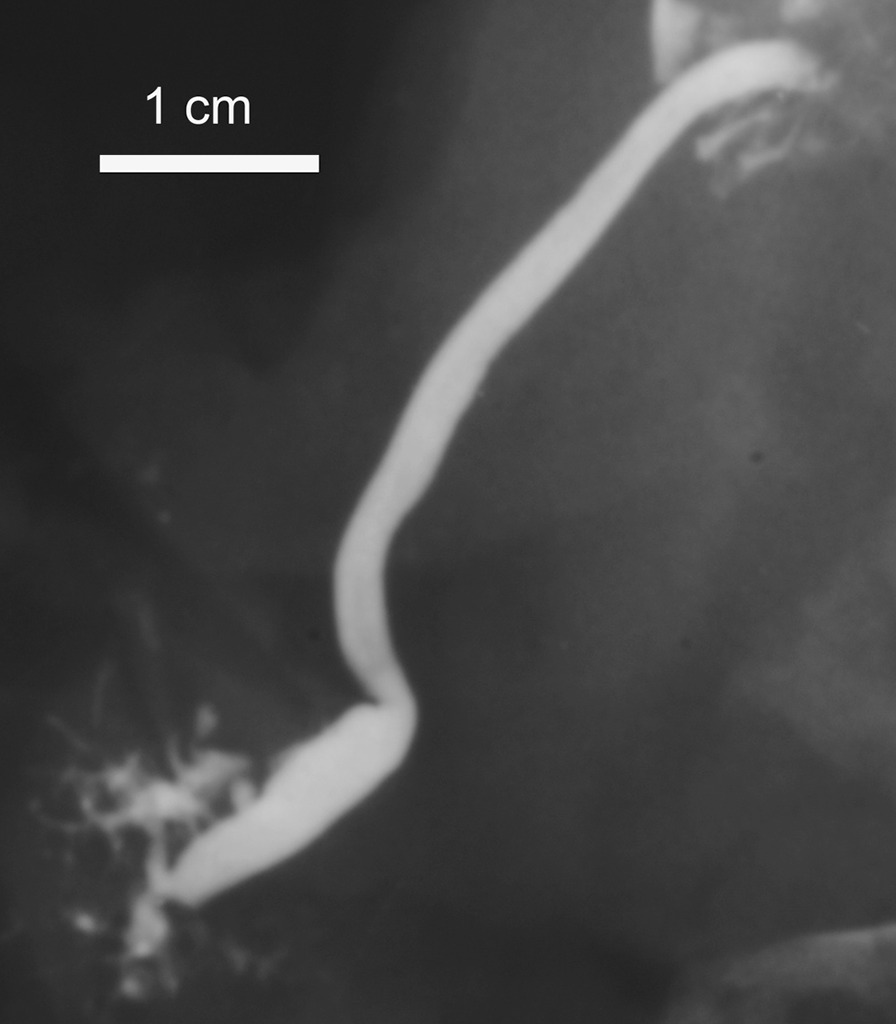
A post-mortem sialogram with pathological findings: intraglandular SMD dilated, side branches “amputated” or dilatated

There were three cases (2 males, one female) with a pathological appearance of the sialography and/or pancreatography (Figs. 3, 4). These cases were excluded from the study.

Further, there were another three cases with insufficient filling of the salivary glands, in two cases it was the right gland and in one the left gland. This missing data underwent casewise deletion in statistical analyses.

### Statistical analysis

The data underwent statistical analyses with the aid of Statistica 64-bit v. 13.5.0.17 (TIBCO Software Inc. 2018), including the Power Analysis, Sample Size Calculation, Shapiro–Wilk W test for normality of distribution, Pearson correlation coefficient for continuous variables, and Spearman rank order correlation or Fisher exact test for categorical variables. Sample size of 39 matched pairs was required to detect a 0.5 Rho correlation, with a 90% statistical power and the significance (alpha-error) of 0.05. Comparable recent studies [1] were used for estimating a sample variability.

## Results

### Weight

The weight of the glands was as follows: pancreas 90.6 ± 25.2 g, left submandibular 10.5 ± 2.5 g, and right submandibular 10.3 ± 2.4 g.

### Length and caliber of ducts

The dimensions of the ductal systems in three glands examined are given in Tables 1 and 2. The entire salivary duct length is around 35% of the MPD length and their maximal caliber is 2/3 of the caliber of the MPD. If only the intraglandular length of the salivary ducts was taken into account, it was less than a half of the whole duct’s length. All the dimensions presented a normal distribution, permitting the correlation analyses.

**Table 1:** Pancreatic and submandibular duct dimensions

	MPD length (mm)	MPD caliber (mm)	SMD length (mm)		SMD caliber (mm)	
			R	L	R	L
Mean ± SD	172.3 ± 31.1	3.7 ± 2.1	63.8 ± 10.2	61.8 ± 8.9	2.6 ± 0.5	2.3 ± 0.5
Min–max	125.0–257.3	0.9 ± 5.4	43.9–82.1	43.4–77.6	1.5–3.5	1.2–3.5
S–W W; *p*	0.948; 0.07	0.965; 0.27	0.974; 0.54	0.962; 0.22	0.974; 0.52	0.970; 0.39

*MPD* main pancreatic duct, *SMD* submandibular duct, *S–W W* Shapiro–Wilk normality test, *p* probability

**Table 2:** Submandibular duct dimensions (intraglandular)

	SMD intraglandular length (mm)	
	R	L
Mean ± SD	29.4 ± 5.4	28.5 ± 5.8
Min–max	20.0–39.6	15.5–38.2
S–W W; *p*	0.957; 0.17	0.961; 0.17

Abbreviations: as in Table 1

The dimensions of the MPD and SMD underwent correlation analyses. The total length of the duct presented no correlation between the pancreas and the SM gland, both on the right and on the left: r = 0.1620 (*p* = 0.338), and r = 0.2184 (*p* = 0.188), respectively. On the other hand, when only the intraglandular portion of the salivary duct was compared to the MPD, there was a significant correlation, both for the right and the left gland: r = 0.3616 (*p* = 0.028), and r = 0.5950 (*p* = 0.000), respectively. As for the mutual correlation between the duct length of the left and the right salivary gland, it was at significant level both for the whole and for the intraglandular length: r = 0.5705 (*p* = 0.000), and r = 0.3286 (*p* = 0.050), respectively.

The maximal caliber of the MPD vs SMD did not show statistical significance, either for the right, or the left salivary gland: r = 0.3109 (*p* = 0.061), and r = 0.1390 (*p* = 0.405). On the other hand, the mutual correlation between the left and the right SMD was significant: r = 0.6833 (*p* = 0.000). The maximal caliber of the SMD was exclusively found extraglandularly.

### Side branches

The descriptive statistics for the MPD and SMD side branches are given on Table 3. When correlating the pancreatic side branches to the ones in the right gland, there was no significance (R = 0.3105, *p* = 0.061). On the contrary, there was a highly significant correlation with the left gland (R = 0.4364, *p* = 0.006), and mutually between the right and the left gland (R = 0.5155, *p* = 0.001).

**Table 3:** Number of primary side branches of the pancreas and salivary glands

	MPD side branches	SMD side branches	
		R	L
Median (SD)	37 (7.6)	9 (2.5)	9 (2.5)
Min–max	19–52	4–14	3–15
S–W W; p	0.964; 0.24	0.970; 0.40	0.966; 0.30

Abbreviations: as in Table 1

### Tortuosities

**Fig. 5:**
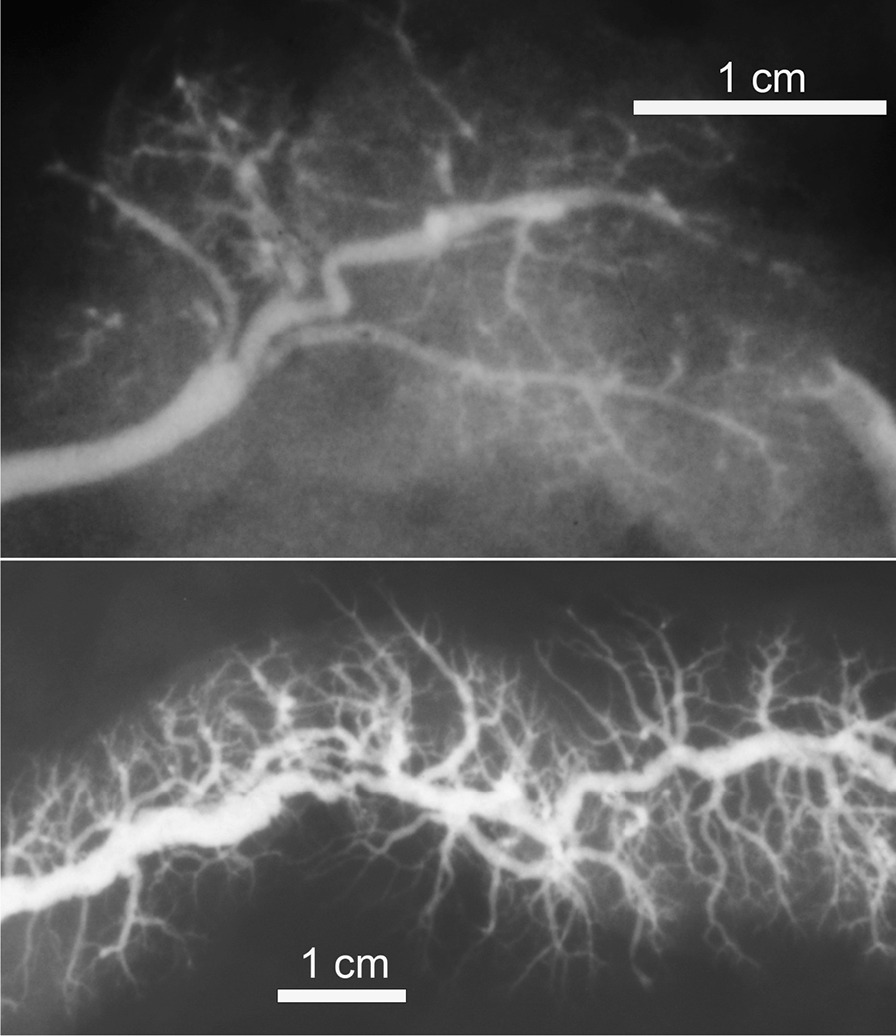
Kinks and tortuosities in the SMD (upper) and MPD (lower)

The tortuosities were observed in 21/39 of MPD, 12/37 of right SMD, and in 9/38 of left SMD (Fig. 5). For this feature, the Fisher exact test presented no association between the MPD and both the right and the left SMD (*p* = 0.049 and *p* = 0.062, respectively). On the other hand, a correlation was observed between the left and the right SMD (*p* = 0.279). The normal “genu” of the SMD, winding around the posterior border of the mylohyoid muscle, was not considered as a tortuosity.

### Accessory ducts/lobes

**Fig. 6:**
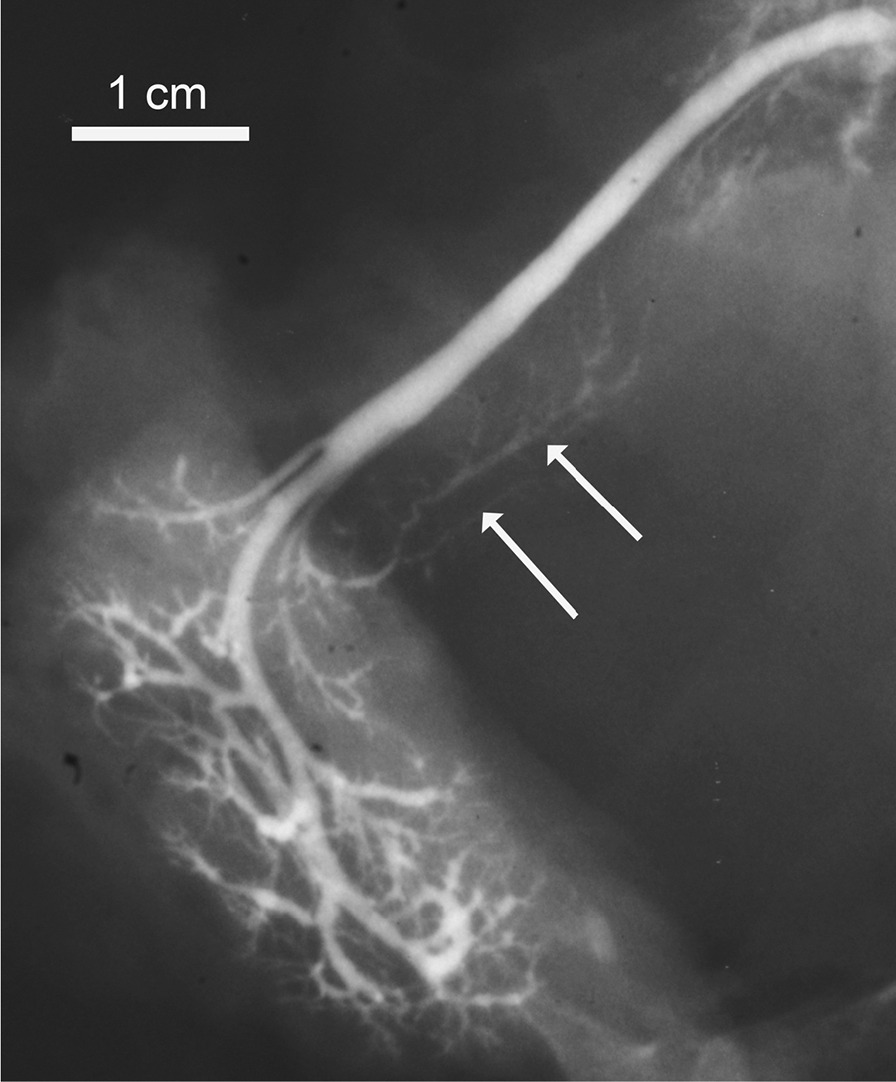
Accessory salivary lobe (arrows) joining the SMD

The search for accessory submandibular ducts/lobes revealed 3 such cases on the right, 2 cases on the left and 2 bilateral (Fig. 6). The homologous structures were not found in the pancreases.

## Discussion

The correlation between different human organs has been studied by means of automatic anatomy recognition, with model objects classified as sparse, non-sparse and hybrid [13]. The delineation and recognition were easiest for non-sparse, i.e. compact, blob-like objects or entities. We can say that the objects of our study, the SM glands and pancreas belong to such a group. The mentioned study by Udupa et al. [13] correlated the size among different objects within the thorax, the abdomen and the neck, but not mutually the soft tissue entities from different regions. Moreover, our study went into finer detail regarding the complete ductal systems of the SM and pancreas gland. The weight of the salivary glands and pancreas, which we measured, were within the normal limits reported previously [14, 15].

The projection images of the entire ductal system of pancreas can be achieved via endoscopic retrograde or magnetic resonance cholangiopancreatography (ERCP and MRCP). The non-invasive MRCP gives an image of lesser quality, and ERCP is thus considered to be the reference method [16]. In cases of equivocal and mild pancreatitis, the appearance of abnormal side branches is highly important for early diagnosis [17]. Likewise, the ductal properties of the salivary glands can be achieved by conventional, digital subtraction or MRI sialography [9, 18].

Our methodology has focused both on the main duct and the side branches, as acinar destruction accompanied by fibrosis is a joint property of chronic inflammatory disease both for the pancreas and the salivary glands [19], and the entire size of the gland itself is a questionable parameter in discriminating the healthy and the diseased organ [9, 20, 21].

It has been noted that MPD and SMD anatomical features such as duct length and caliber can be implied in etiopathogenesis of different diseases, such as pancreatitis, sialolithiasis and sialadenitis [17, 20]. Our first analysis applied to the length of the MPD and SMD. The two organs differ in the fact that more of a half of the SMD is situated extraglandularly, which is not the case with pancreas. The lack of correlation between the MPD and the full-length SMD can be attributed to the large range of variation of this parameter; when only the SMD intraglandular portion was taken into account, the correlation appeared as significant. A retrospective study of digital subtraction sialograms found a mean value for SMD length of 58 mm [20], somewhat inferior to our measurements. The differences can be explained by a higher pressure of contrast injection in post-mortem specimens.

The caliber (internal diameter) of the MPD and the SMD is a valuable morphometric factor on which one can base the algorithm in clinical evaluation. It can be measured at different locations, such as head, body or tail in pancreas, or proximal, middle and distal third of the SMD [20, 22]. Instead, we opted for one maximal caliber as being a more comparable feature. The median value for the MPD maximal caliber was close to the upper limit, but still within the normal ranges for the disease-free gland [22]. In case of the SMD, the median maximal calibers we measured (2.6 mm and 2.3 mm) were also within the upper limits given by Horsburgh and Massoud [20], despite the methodological differences, i.e. post-mortem vs. digital subtraction sialography. However, in our study there was no correlation between the MPD and SMD maximal calibers.

Although the majority of interest within the two glands’ morphology has been focused on their principal excretory ducts [20, 23], the MPD and SMD primary side branches have also been the objective of studies [9, 17, 24]. The vast majority of side branches open on each side of the main duct with very few on anterior and posterior walls [25]. In our study, even those few ducts were visible as they do not take a geometrically ideal orthogonal course. It has been shown that irregularities of peripheral ducts, such as cystic dilatations, ectasia and lack of opacification are signs of initial and mild inflammation. On the other hand, it must be underlined that non-invasive imaging has a lower rate of presenting these side branches, e.g. MRCP vs ERCP, or MR sialography vs. conventional or digital subtraction sialography [20, 23, 26]. Our post-mortem ductography achieved the complete arborization pattern, except in three submandibular glands. However, the correlation of side branches’ number was indeterminate, pancreas vs. left gland showed high significance, but pancreas vs. right gland did not, although it was close to the upper limit of significance. One of the plausible explanations for this can be found in the embryology of the MPD, which derives from the fusion of the ducts from ventral and dorsal pancreatic buds. The major part of MPD is derived from dorsal bud that composes the left (body-tail) part of postnatal pancreas.

Apart from the duct dimensions (length and caliber), the pattern of its course is also of diagnostic and therapeutic value. For instance, a tortuous MPD can pose technical difficulties for stent insertion [27], and a tortuous salivary duct can compromise balloon dilatation [28]. With respect to the presence of ductal tortuosities, our study did not reveal an association between the pancreas and the salivary glands. Therefore, this feature can be ascribed to individual variability.

The left–right comparison of paired organs has also included the submandibular glands, mainly by measuring the cross-sectional or planimetric area occupied by the gland [1, 29]. The detailed morphometry of the SMD ducts [20] did not, however, include the question of symmetry. Therefore, we carried out these analyses with regard to all the morphometric parameters observed (length, caliber, tortuosity, side branches of the SMD) and found full symmetry between the two sides.

Last but not least, we have observed cases with accessory submandibular ducts and lobes, not as an independent glandular structure [30], but mostly in the forms of outgrowths of the principal glands. This is in line with the embryology of the gland, where there is interaction between the endoderm and the neighboring mesenchyme, resulting in budding of the glands [19]. Similar finding was not found in pancreases.

The clinical impact of our study is based on the premise that both glands, salivary and pancreas, can suffer from autoimmune or alcoholic pathology. Co-existent sialography changes have been reported in patients with chronic pancreatitis [9, 10]. Hence, clinical approach in those two types of pancreatitis (but not biliary) can benefit from evaluation of the salivary glands, particularly in the view of their incidence accounting for the majority of pancreatitis cases per se. A follow-up of chronic pancreatitis patients should include the assessment of the salivary glands because of their role in swallowing, neutralization of residual acid in the esophagus, oral health and taste sensation [31]. Further, pathology of salivary glands has been reported as a marker of a severe stage of the disease [32].

### Limitations

This study has three limitations. One concerns the material sample with a predominance of males, and the other is a single measurement of dimensions rather than repetitive to obtain reproducibility of results. The third one is lack of body metrics (height, weight) which can have an influence on the morphometry of analyzed organs. Our study used the pairwise correlative analyses, which reduces such an influence. Additionally, our sample included subjects of younger middle age groups. An autopsy retrospective study has presented correlation of different organ weights with the subjects’ age, body weight and height [33], defining the age of 19 years as a cut-off point after which there are only individual variations, but no more general increases in organ weight.

## Conclusions

Our study has given valuable morphometric data regarding the pancreas and the submandibular glands. On the other hand, the correlation of these parameters rests partly inconclusive, while length of the MPD and SMD correlate well, the caliber of the ducts does not, and the number of side branches only partly. Therefore, we look forward to a similar study set out in a clinical environment.

## Data Availability

The datasets used and/or analysed during the current study are available from the corresponding author on reasonable request.

## Abbreviations

MPD: Main pancreatic duct (Wirsung’s duct)
SMD: Submandibular duct (Wharton’s duct)
SM: Submandibular
S–W W: Shapiro–Wilk normality test
*p*: Probability

## References

[1] Stimec BV, Rakocevic Z, Ignjatovic D, Fasel JHD (2018). Planimetric correlation between the submandibular glands and the pancreas: a postmortem ductographic study. Anat Sci Int.

[2] Rela, M, Reddy SM. Pancreas. In: Standring S (ed) Gray’s anatomy. The anatomical basis of clinical practice, 41st edn. p. 1179–87;2016. ISBN 978-0-7020-5230-9

[3] Quirós-Terrón L, Arráez-Aybar LA, Murillo-González J, De-la-Cuadra-Blanco C, Martínez-Álvarez MC, Sanz-Casado JV, Mérida-Velasco JR (2019). Initial stages of development of the submandibular gland (human embryos at 5.5–8 weeks of development). J Anat.

[4] Gorjup E, Danner S, Rotter N, Habermann J, Brassat U, Brummendorf TH, Wien S, Meyerhans A, Wollenberg B, Kruse C, von Briesen H (2009). Glandular tissue from human pancreas and salivary gland yields similar stem cell populations. Eur J Cell Biol.

[5] Matsuzaki T, Tajika Y, Ablimit A, Aoki T, Hagiwara H, Takata K (2004). Aquaporins in the digestive system. Med Electron Microsc.

[6] Delporte C (2014). Aquaporins in salivary glands and pancreas. Biochim Biophys Acta.

[7] Badea AF, Lupsor Platon M, Crisan M, Cattani C, Badea I, Pierro G, Sannino G, Baciut G (2013). Fractal analysis of elastographic images for automatic detection of diffuse diseases of salivary glands: preliminary results. Comput Math Methods Med.

[8] Pajevic M, Aleksic M, Golic I, Markelic M, Otasevic V, Jankovic A, Stancic A, Korac B, Korac A (2018). Fractal and stereological analyses of insulin-induced rat exocrine pancreas remodelling. Folia Morphol (Warsz).

[9] Frulloni L, Morana G, Bovo P, Mansueto GC, Vaona B, Di Francesco V, Procacci C, Cavallini G (1999). Salivary gland involvement in patients with chronic pancreatitis. Pancreas.

[10] Kamisawa T, Tu Y, Egawa N, Sakaki N, Inokuma S, Kamata N (2003). Salivary gland involvement in chronic pancreatitis of various etiologies. Am J Gastroenterol.

[11] Stimec BV, Draskic M, Fasel JH (2010). Cadaver procurement for anatomy teaching: legislative challenges in a transition-related environment. Med Sci Law.

[12] Republic of Serbia, Law on Health Protection. Chapter XVIII—Establishing time and cause of death and autopsy of deceased persons, Article 206; and Chapter XIX—Procurement of bodies of deceased persons for the purpose of practical medical education, Articles 210 through 216. Official Gazette 2019. https://www.pravno-informacioni-sistem.rs/SlGlasnikPortal/eli/rep/sgrs/skupstina/zakon/2019/25/2. Accessed 19 Oct 2021.

[13] Udupa JK, Odhner D, Zhao L, Tong Y, Matsumoto MM, Ciesielski KC, Falcao AX, Vaideeswaran P, Ciesielski V, Saboury B, Mohammadianrasanani S, Sin S, Arens R, Torigian DA (2014). Body-wide hierarchical fuzzy modeling, recognition, and delineation of anatomy in medical images. Med Image Anal.

[14] de Paula F, Teshima THN, Hsieh R, Souza MM, Nico MMS, Lourenco SV (2017). Overview of human salivary glands: highlights of morphology and developing processes. Anat Rec.

[15] Caglar V, Songur A, Yagmurca M, Acar M, Toktas M, Gonul Y (2014). Study of volume, weight and size of normal pancreas, spleen and kidney in adults autopsies. Forensic Med Anat Res.

[16] Evrimler S, Swensson JK, Soufi M, Tirkes T, Schmidt CM, Akisik F (2021). Wirsungocele: evaluation by MRCP and clinical significance. Abdom Radiol (NY).

[17] Sai JK, Suyama M, Kubokawa Y, Watanabe S (2008). Diagnosis of mild chronic pancreatitis (Cambridge classification): comparative study using secretin injection-magnetic resonance cholangiopancreatography and endoscopic retrograde pancreatography. World J Gastroenterol.

[18] Kalinowski M, Heverhagen JT, Rehberg E, Klose KJ, Wagner HJ (2002). Comparative study of MR sialography and digital subtraction sialography for benign salivary gland disorders. AJNR Am J Neuroradiol.

[19] Rakonczay Z, Vág J, Földes A, Nagy K, Nagy Á, Hegyi P, Varga G (2014). Chronic inflammation in the pancreas and salivary glands–lessons from similarities and differences in pathophysiology and treatment modalities. Curr Pharm Des.

[20] Horsburgh A, Massoud TF (2013). The salivary ducts of Wharton and Stenson: analysis of normal variant sialographic morphometry and a historical review. Ann Anat.

[21] Treiber M, Einwächter H, Phillip V, Wagenpfeil S, Schmid RM, Lersch C (2016). Is the size of the pancreas useful in diagnosing chronic pancreatitis? An ultrasound based, retrospective study. Pancreatology.

[22] Eloubeidi MA, Luz LP, Tamhane A, Khan M, Buxbaum JL (2013). Ratio of pancreatic duct caliber to width of pancreatic gland by endosonography is predictive of pancreatic cancer. Pancreas.

[23] Dugic A, Nikolic S, Mühldorfer S, Bulajic M, Pozzi Mucelli R, Tsolakis AV, Löhr JM, Vujasinovic M (2020). Clinical importance of main pancreatic duct variants and possible correlation with pancreatic diseases. Scand J Gastroenterol.

[24] Ito T, Ikeura T, Tanaka T, Mitsuyama T, Miyoshi H, Shimatani M, Uchida K, Takaoka M, Okazaki K (2020). Magnetic resonance cholangiopancreatography findings in early chronic pancreatitis diagnosed according to the Japanese Diagnostic Criteria. Pancreatology.

[25] Sahni D, Jit I, Harjeet A (2001). Gross anatomy of the pancreatic ducts in north Indians. Trop Gastroenterol.

[26] Varghese JC, Thornton F, Lucey BC, Walsh M, Farrell MA, Lee MJ (1999). A prospective comparative study of MR sialography and conventional sialography of salivary duct disease. AJR Am J Roentgenol.

[27] Freeman ML, Overby C, Qi D (2004). Pancreatic stent insertion: consequences of failure and results of a modified technique to maximize success. Gastrointest Endosc.

[28] Makdissi J, Feinberg L, Roy A (2017). Is there a role for ultrasound-guided balloon sialoplasty technique in salivary gland structures?. Dentomaxillofac Radiol.

[29] Heo MS, Lee SC, Lee SS, Choi HM, Choi SC, Park TW (2001). Quantitative analysis of normal major salivary glands using computed tomography. Oral Surg Oral Med Oral Pathol Oral Radiol Endod.

[30] Nayak SB (2018). Accessory submandibular salivary gland forming a "horseshoe" with the main submandibular salivary gland: a unique variation. J Craniofac Surg.

[31] Dutta SK, Dukehart M, Narang A, Latham PS (1989). Functional and structural changes in parotid glands of alcoholic cirrhotic patients. Gastroenterology.

[32] Fukui T, Okazaki K, Yoshizawa H, Ohashi S, Tamaki H, Kawasaki K, Matsuura M, Asada M, Nakase H, Nakashima Y, Nishio A, Tsutomu C (2005). A case of autoimmune pancreatitis associated with sclerosing cholangitis, retroperitoneal fibrosis and Sjögren’s syndrome. Pancreatology.

[33] Ogiu N, Nakamura Y, Ijiri I, Hiraiwa K, Ogiu T (1997). A statistical analysis of the internal organ weights of normal Japanese people. Health Phys.

